# Effect of Prolonged Frozen Storage on the In Vitro Digestion of Minced Pork: Insights from Protein Structural Changes

**DOI:** 10.3390/foods15020329

**Published:** 2026-01-16

**Authors:** Yingying Zhu, Shijing Chen, Yafang Ma, Caili Fu, Dejian Huang

**Affiliations:** 1 Food & Medicine Homology Big Health Innovation Consortium, Department of Food Nutrition and Test, Suzhou Polytechnic University, Suzhou 215104, China; 2 Biomedical and Health Technology Platform, National University of Singapore (Suzhou) Research Institute, Suzhou 215128, China

**Keywords:** pork, frozen storage, in vitro digestion, proteomics, protein oxidation

## Abstract

Long-term frozen storage is widely used for pork preservation, yet its impact on protein digestibility remains inadequately explored. This study investigated the effect of frozen storage duration (0, 3, and 12 months) on the changes in digestive properties and protein structure of minced pork during in vitro digestion. With extended freezing, the hardness, chewiness, and shear force of pork significantly increased, while protein digestibility decreased. A confocal laser scanning microscope showed an increase in the particle size of digesta. After 12 months of frozen storage, the digestibility of the pork samples decreased. The extent of reduction reached 11.31% under pepsin digestion and 11.33% under pepsin–trypsin digestion, compared to the fresh samples. Structural analysis indicated that prolonged freezing led to protein denaturation and aggregation, as confirmed by a decrease in α-helix contents, an increase in the β-sheet and random coil structures, and the formation of high-molecular-weight aggregates. These structural alterations hindered protease accessibility, resulting in reduced digestibility. The detrimental effects on protein structure and digestibility became more pronounced with longer frozen storage.

## 1. Introduction

Pork is globally recognized as an important source of animal protein, valued for its nutritional quality and cost-effectiveness [1,2]. However, these nutrient rich compositions make pork highly perishable, requiring proper storage methods. Freezing is a well-established technique for extending the shelf-life of meat products by suppressing microbial growth and decelerating biochemical reactions [3,4]. Due to its effectiveness, freezing plays a critical role in national meat reserves, market supply management, and the industrial-scale preservation of raw materials [5]. Data from China’s Ministry of Agriculture and Rural Affairs (2024) indicated that pork production reached 57.06 million tons in 2024, approximately 20% of this production is subjected to freezing and storage for national reserves and industrial-scale preservation, with the freezing duration potentially extending up to one year. As consumer demand shifts from basic supply assurance to expectations of “safe, tasty, nutritious, and healthy” products, the nutritional quality and safety of frozen meat have attracted growing attention [6].

Existing studies indicate that freezing imparts mechanical forces through ice crystal nucleation and growth, compromising the hydrogen bonds between protein side chains and bound water, which alters physicochemical and structural properties [7], while ionic stress and pH shifts contribute to myofibrillar denaturation [8]. These modifications lead to undesirable effects such as reduced tenderness [9], discoloration, protein aggregation [10], and microstructural deterioration [11]. These changes diminish both the sensory appeal and the potential nutritional quality of the meat, primarily due to protein denaturation.

Current research on frozen meat has predominantly focused on flavor compounds and lipid oxidation [12], as well as freezing rates [13] and thawing techniques, such as electrostatic fields [14] and ultrasound-assisted methods [15]. However, the specific effects of long-term frozen storage on protein structural evolution and subsequent proteolytic digestibility remain inadequately explored, especially beyond 12 months. Protein digestibility is considered a crucial factor in assessing the nutritional value of meat [16]. While thermal processing (e.g., boiling, frying) and novel technologies (e.g., high-pressure treatment, ultrasound) have been shown to alter protein conformation and digestibility [17,18,19], the long-term impact of freezing on enzymatic hydrolysis is still not well characterized. In particular, the roles of cryo-denaturation and oxidative cross-linking influence protein accessibility require further investigation. It is hypothesized that freezing-induced protein modifications—such as aggregation, conformational shifts, and oxidation—may sterically hinder proteolytic cleavage sites or disrupt enzyme–substrate binding, thereby reducing digestibility.

Recent study revealed that prolonged freezing induces structural changes in pork myosin, which may impede enzymatic breakdown [20]. Nonetheless, comprehensive studies correlating frozen storage duration with meat quality deterioration, protein structural alterations, and in vitro digestibility outcomes are still scarce. Therefore, systematic investigation of how extended freezing influences protein integrity and digestive behavior is crucial for advancing meat preservation strategies and safeguarding nutritional value throughout supply chains. This study aims to evaluate how frozen storage periods (0, 3, 12 months) impact pork quality attributes and elucidate the underlying mechanisms between protein structural changes and digestive properties.

## 2. Materials and Methods

### 2.1. Sample Preparation

*Longissimus dorsi* muscle from six pigs was obtained from Jiangsu Food Co., Ltd. (Huaian, China) and transported under cold chain conditions. Upon arrival at the laboratory, removed visible fat and connective tissue from the muscle, cut into 2 × 2 × 2 cm cubes for future use. The muscle was then ground using a meat grinder (model: JR05SD-300, Supor Co., Ltd., Hangzhou, China) at a speed of 3800 rpm with a standard four-bladed rotating cutting knife for 30 s to produce minced meat and then passed through a stainless steel sieve with 4 mm pores. The sieved minced meat was collected to ensure uniform texture and remove incompletely minced tissue fragments and then was subsequently shaped into patties with a diameter of 10 cm and a thickness of 1.5 cm. Each patty was randomly but carefully placed in a disposable, food-grade polyethylene (PE) self-sealing bag (15 × 20 cm, 0.08 mm thickness), from which air was gently expelled by pressing the bag flat from the bottom upward before sealing. The samples were stored at −18 ± 1 °C for different frozen storage time. The frozen storage time was treated as a fixed-effect factor with three levels (0, 3, and 12 months). Prior to analysis, the frozen patties were thawed in a refrigerator at 4 °C for 24 h.

### 2.2. Color Analysis

According to the method described by Niu Yuge [21], the frozen pork patties which had been thawed in a refrigerator at 4 °C for 24 h as mentioned above were taken out, were allowed to equilibrate to room temperature, and the surface water was blotted dry with absorbent paper. The color parameters of pork samples, including lightness (L*), redness (a*) and yellowness (b*), were quantified by spectrophotometer (YS3010, 3NH Technology Co., Ltd., Shenzhen, China) after zeroing by whiteboard. The port size is 8 mm, the observer is 2 degrees, and the spectral component was excluded. Each group of samples was replicated 5 times, and 3 groups of data were selected to take the average value.

### 2.3. Texture Profile Analysis

Using a slightly adapted version of a previously established method [22], the hardness, chewiness, and shear force of heated pork samples were evaluated with a Texture Analyzer (Universal TA, Shanghai, China). Each sample was uniformly cut into 2 × 2 × 2 cm cubes and tested using a P/50 flat-faced cylindrical probe. The compression test was performed at a speed of 2.0 mm/s (crosshead speed). The settings included a test interval of 5 s, a measurement mode of 40% deformation, and pre-test of 2.0 mm/s and post-test speeds of 5.0 mm/s. Additionally, shear force was measured in Newtons using a blade-equipped Texture Analyzer (from the same manufacturer), set to a test speed of 4.0 mm/s and a maximum force threshold of 30 g.

### 2.4. Juice Loss

Juice loss, including centrifugal loss, thawing loss and cooking loss, was evaluated according to established methodologies. Centrifugal loss was measured following an adapted procedure based on Salvador et al. [23]. Briefly, pork samples were thawed and weighed (m_1_), then centrifuged at 10,000 rpm for 20 min in a centrifuge and weighed again (m_2_). The centrifugal loss was calculated as follows:

(1) $$Centrifugal\;loss\left(\%\right)=\frac{m_{1}-m_{2}}{m_{1}}\times 100$$

Thawing loss and cooking loss were quantified as described by Zhu et al. [24]. Briefly, pork samples were weighed before thawing (m_3_), then thawed and weighed (m_4_). The thawing loss was calculated as follows:

(2) $$Thawing\;loss\left(\%\right)=\frac{m_{3}-m_{4}}{m_{3}}\times 100$$

The thawed samples were placed in cooking bags and heated in a 72 °C water bath until the core temperature reached 70 °C. Subsequently, the samples were cooled to room temperature and weighed (m_5_). Cooking loss was calculated as follows:

(3) $$Cooking\;loss\left(\%\right)=\frac{{m_{4}-m}_{5}}{m_{4}}\times 100$$

### 2.5. In Vitro Digestion

Prior to digestion, the thawed samples were placed in cooking bags and heated in a 72 °C water bath until the core temperature reached 70 °C for future use. The compositions of the simulated digestion fluids were listed in Supplementary Tables S1–S3. Methodology of the in vitro digestion was adapted from Lin et al. with modifications [25]. Briefly, 2.0 g of sample was homogenized in 10 mL of ultrapure water, 10 mL of simulated oral fluid (pH = 7.0) was added, followed by incubation at 37 °C with constant agitation (150 rpm, 2 min) to simulate oral digestion. Subsequently, gastric digestion was subsequently commenced with the introduction of 20 mL of simulated gastric fluid (pH 2.0) to each sample. The mixtures were subjected to gastric digestion by incubating at 37 °C with orbital shaking (150 rpm, 2 h). To halt enzymatic reactions, the samples were heated in a boiling water bath (100 °C) for 10 min. Subsequently, 40 mL of simulated intestinal fluid (pH 7.0) was mixed with the gastric digesta. This mixture was incubated for another 2 h under the aforementioned shaking parameters to achieve full hydrolysis. A final heat treatment (100 °C, 10 min) was applied to prepare the intestinal digesta for further analysis.

#### 2.5.1. Protein Digestibility

The gastric and intestinal digesta were cooled to room temperature, then centrifuged (7500 *g*, 4 °C, 15 min) for removing undigested proteins. The supernatant was then subjected to a ten-fold dilution with ultrapure water. Protein levels were quantified via the bicinchoninic acid (BCA) assay [26], which measured soluble peptides and small proteins released during digestion. Specifically, the reaction system consisted of 10 μL of diluted sample mixed with 200 μL of BCA working solution in a 96-well microplate, which was subsequently maintained at 37 °C for a 30 min incubation period. A BioTek Synergy HTX reader (Agilent, Santa Clara, CA, USA) was used for the optical density measured under 562 nm. The final protein content was quantified based on a standard curve, with all measurements performed in triplicate.

Protein digestibility was calculated using the following formula:

(4) $$Protein\;digestibility\left(\%\right)=\frac{C*V*10}{M}\times 100$$

where C was the protein concentration (mg/mL) of the digestive product after deducting the blank (buffer and digestive enzyme) measured by BCA method; V was the volume of digestive product, and M was the protein mass of the sample before digestion, was measured by the Kjeldahl method. The digestibility calculated by the end point of digestion was the simulated gastric digestibility of protein in vitro, and the digestibility calculated by the end point of intestinal digestion was the digestibility calculated by the end of intestinal digestion.

#### 2.5.2. Free Amino Acid

An automatic amino acid analyzer (L-8900, Hitachi Ltd., Tokyo, Japan) was employed to determine the free amino acid (FAA) content in pork digesta [25]. Samples of digested material were centrifuged (10,000 rpm, 15 min) to obtain a supernatant. Proteins in the supernatant were then precipitated by adding 5% sulfosalicylic acid at 1:4 ratio. The mixture was subjected to further centrifugation (18,000 rpm, 30 min) to obtain the supernatant, then membrane filtered (0.45 μm) in preparation for analysis. The concentration of FAA in the supernatant was determined by the amino acid analyzer and expressed as C (mg/100 mL of the prepared sample). This value was converted to express the FAA content relative to the mass of the original pork sample using the following calculation to account for dilutions and digest volumes:

(5) $$FAA\;(mg/g)=\frac{C\times 1.25\times V_{total}}{100\times M}$$

where C was the FAA concentration from the analyzer (mg/100 mL), 1.25 was the dilution factor from the sulfosalicylic acid precipitation step, V_total_ was the total volume of the final intestinal digest, and M was the mass of the pork sample used for digestion.

### 2.6. Digestive Samples Microstructure

Confocal laser scanning microscope (CLSM, LSM 800, Carl Zeiss AG, Oberkochen, Germany) was employed to observe the microstructural features of protein particles in the digested samples following an adapted analytical procedure [27]. Briefly, 40 μL of Nile blue solution (1% *w*/*v* in isopropanol) was added to 1.0 mL aliquots of both gastric and intestinal digesta (0.5 mg/mL). The mixtures were incubated in the dark for 5 min to facilitate sufficient dye–protein interaction. Subsequently, 5 μL aliquots of each stained sample were deposited onto microscope slides for imaging. Protein components were specifically visualized using a 633 nm helium-neon laser for excitation under a ×40 objective lens.

### 2.7. SDS-PAGE

Sodium dodecyl sulfate-polyacrylamide gel electrophoresis, shortly SDS-PAGE, was introduced using a 12% resolving gel (ET12012Gel, ACE Biotechnology Co., Ltd., Nanjing, China) to determine the molecular weight distribution of proteins, following an optimized protocol adapted from conventional methods described by Bai et al. [22]. Digestion samples (2 mg/mL protein) were combined with 5× Laemmli loading buffer (WB2001, NCM Biotech Co., Ltd., Suzhou, China) at a volume ratio of 4:1 and subsequently heated at 100 °C for 15 min to achieve denaturation. Electrophoresis was measured by Mini-PROTEAN Tetra system (Bio-Rad, Hercules, CA, USA), loading 20 μL of denatured samples alongside 5 μL of a pre-stained protein ladder (PR1920, Solarbio Science & Technology Co., Beijing, China) into separate lanes. Following separation, Coomassie Brilliant Blue R-250 staining solution (P0071A-1, Beyotime Biotechnology Co., Ltd., Shanghai, China) was applied to the gel, which was incubated for 30 min with gentle agitation. The excess stain was then removed by destaining solution (P0071A-2, Beyotime Biotechnology Co., Ltd., Shanghai, China) until the protein bands were clearly visible against a clear background.

### 2.8. FTIR

FTIR (Fourier transform infrared) spectroscopy was employed to characterize the secondary changes in gastric digestive products. The freeze-dried samples were homogenously blended with potassium bromide at a ratio of 1:100 (*w*/*w*) and then subjected to FTIR analysis. Spectral acquisition was performed by Nicolet iS50 spectrometer (Thermo Fisher Scientific, Waltham, MA, USA). Spectral data were acquired at ambient temperature over the wave number range of 400–4000 cm^−1^ with 4 cm^−1^ resolution, accumulating 64 scans per spectrum to ensure adequate signal-to-noise ratio. The obtained spectral data were processed with OMNIC9.2 (software, Thermo Fisher Scientific, Waltham, MA, USA) for baseline correction and smoothing. The amide I region (1600–1700 cm^−1^) was subjected to Gaussian curve fitting in Peak Fit 4.12 (Systat Software, Inc., San Jose, CA, USA) to quantify the protein secondary structure.

### 2.9. Data Analysis

The experimental design was a completely randomized design. The study was conducted with six independent biological replicates. Each biological replicate consisted of a patty processed and analyzed independently throughout the entire protocol, from frozen storage to in vitro digestion and subsequent assays. For each biological replicate, analytical measurements were performed in triplicate, with the average value used for statistical analysis. All data were illustrated in mean ± SD (standard deviation). Statistical analyzes were conducted with Statistix 8.1 using one-way ANOVA followed by Tukey’s HSD (honestly significant difference) test, with a significance threshold of *p* < 0.05. The statistical analysis was based on the following mathematical model:

(6) $$Y_{ij}=\mu +\alpha_{i}+\varepsilon_{ij}$$

where Y_ij_ was the observed response for the jth biological replicate (j = 1, 2, 3, 4, 5, 6) within the ith storage time level; μ was the overall population mean; α_i_ was the fixed effect due to the ith level of frozen storage time (i = 0, 3, 12 months); ε_ij_ was a random deviation of the jth individual of group i from μ + α_i_.

All figures were produced in Origin 2021 (OriginLab, Inc., Northampton, MA, USA) using consistent formatting settings.

## 3. Results and Discussion

### 3.1. Frozen Storage Induced Color Change

**Figure 1:**
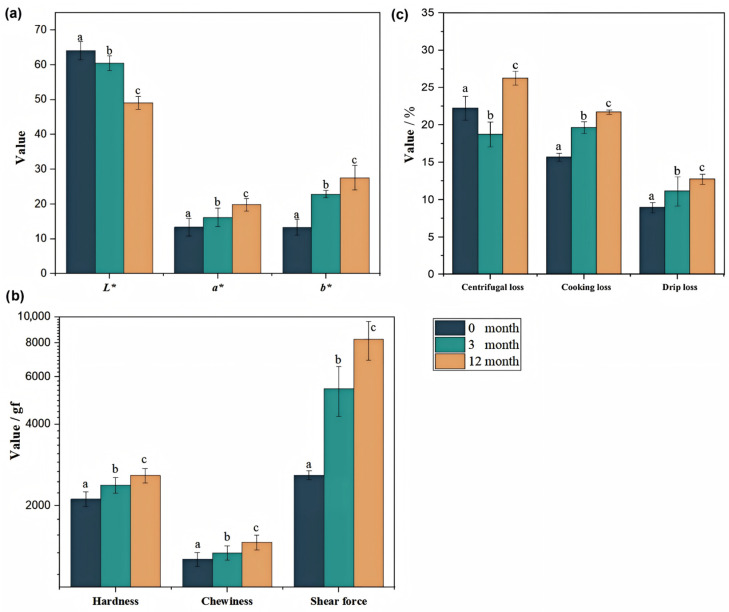
Effect of freezing storage time on pork quality. (**a**) color; (**b**) texture profile; (**c**) juice loss. Note: Means with standard error were indicated, significant differences (*p* < 0.05) among treatments were indicated by different superscript letters (n = 6).

The L* value denotes lightness, with lower and higher values representing darker and lighter colors, respectively. The a* values represent the red-green axes, while b* stands for yellow-blue axes, characterized by positive and negative values, respectively [28]. As shown in Figure 1a, the color parameters of pork samples were significantly influenced (*p* < 0.05) by frozen storage periods. As freezing time increased, the L* (64.09 ± 2.60, 60.43 ± 2.10 and 49.08 ± 1.82 in samples of 0, 3, and 12 months, respectively) and b* values (13.35 ± 2.25, 22.86 ± 1.03, and 27.57 ± 3.56 in samples of 0, 3, and 12 months, respectively) showed a progressive increase, while the a* value (13.37 ± 2.56, 9.19 ± 2.71, and 5.79 ± 1.80 in samples of 0, 3, and 12 months, respectively) decreased. These changes could be attributed to the physiological and oxidative damage incurred during frozen storage. The decrease in L* value resulted from moisture loss and structural damage to muscle fibers from ice crystal formation, which led to a rougher surface and reduced light reflectance [21]. The significant decrease in the a* value was primarily due to the oxidation of myoglobin to metmyoglobin, which imparted a brownish color to the meat. The extended freezing duration accelerated the loss of cellular integrity, thereby increasing the exposure of the pigments to oxygen. Furthermore, free radicals generated by lipid oxidation further exacerbated this change [29]. The increase in b* value with extended freezing was associated with intensified lipid oxidation [30], which produced a substantial quantity of yellow-hued carbonyl compounds, such as aldehydes and ketones. Additionally, non-enzymatic browning reactions, notably the Maillard reaction, may have contributed to the formation of yellow pigments, further amplifying the observed rise in b* values.

### 3.2. Textural Hardening with Extended Frozen Storage

As shown in Figure 1b, the texture properties of pork meat pies, including hardness (2115.17 ± 130.66, 2379.91 ± 154.47 and 2584.70 ± 156.17 in samples of 0, 3, and 12 months, respectively), chewiness (1265.36 ± 75.22, 1337.30 ± 82.83 and 1462.55 ± 92.16 in samples of 0, 3, and 12 months, respectively), and shear force (2524.01 ± 983.00, 5409.87 ± 1138.68 and 8265.85 ± 1355.07 in samples of 0, 3, and 12 months, respectively), were significantly influenced by freezing duration (*p* < 0.05). These changes were attributed to microstructural damage caused by ice crystal formation and growth during freezing, which disrupted muscle fibers and led to the release of intracellular components, including proteins [31]. As freezing was extended, the expansion of ice crystals further compromised muscle integrity and promoted the formation of a more compact tissue structure, thereby elevating hardness [32]. Freezing also induced protein denaturation, particularly in myofibrillar proteins [33]. Extended freezing durations altered the spatial conformation of protein molecules, enhancing intermolecular interactions and promoting the formation of cross-linking bonds; the protein network structure became more dense, further elevating hardness. Concurrently, chewiness and shear force increased significantly with freezing time, indicating that more chewing cycles and greater mechanical force were required to break down the pork, suggesting potential impediments to subsequent gastrointestinal digestion due to reduced efficiency in both mechanical disintegration and enzymatic hydrolysis [34]. The significant increase in hardness and shear force suggested the formation of a tougher, more cross-linked protein network. This textural hardening was anticipated to not only challenge mastication but also impede the subsequent enzymatic hydrolysis in the gastrointestinal tract, potentially leading to reduced digestibility.

### 3.3. Water-Holding Capacity Declined Due to Extended Frozen Storage

The analysis of juice loss, including centrifugal loss, thawing loss, and cooking loss, was shown in Figure 1c. The results revealed a significant deterioration (*p* < 0.05) in the water-holding capacity (WHC) of pork with extended freezing duration. A notable non-linear trend was observed in centrifugal loss. Fresh samples (at 0 months) exhibited a centrifugal loss of 22.22 ± 1.60%, which significantly decreased to 18.72 ± 1.66% after 3 months of freezing. The initial increase implies that short-term freezing can temporarily promote water-holding capacity, which may stem from modest denaturation of myofibrillar proteins and the subsequent formation of a short-lived gel structure capable of trapping water [35]. However, this beneficial effect was reversed with prolonged storage, as the centrifugal loss surged to 26.27 ± 0.92% in the 12-months frozen samples, significantly exceeding all other groups. This indicated that cumulative cryodamage eventually overwhelmed the initial protective mechanisms. A consistent and significant upward trend was observed for both drip loss (8.96 ± 0.68%, 11.14 ± 1.94%, and 12.74 ± 0.67% for 0, 3, and 12 months, respectively) and cooking loss (15.69 ± 0.54%, 19.64 ± 0.80%, and 21.71 ± 0.28% for 0, 3, and 12 months, respectively) with increasing freezing time (*p* < 0.05). The deterioration in WHC was attributed to a combination of physical and chemical changes. The growth of ice crystals mechanically damaged cells and created pores, preventing water retention [7]. The increase in drip loss confirmed that extended freezing promoted the conversion of immobilized water and bound water to free water, which was readily released upon thawing. As freezing duration extended, ice crystals progressively merged and enlarged through Ostwald ripening, forming sharp, large crystals that puncture the sarcolemma and cell membranes, compromising the integrity of muscle tissue. Upon thawing, the damaged cells failed to retain water effectively, leading to significant drip loss. The phenomenon of increased cooking loss in pork due to prolonged freezing duration was closely associated with complex mechanisms involving cellular structural damage, protein denaturation, and moisture migration. Prolonged cold induced protein denaturation and aggregation, hindering hydrogel formation [10]. Extended freezing durations further accelerated lipid oxidation, generating reactive compounds such as malondialdehyde (MDA), which attacked sulfhydryl groups and amino acid side chains in proteins, exacerbating cross-linking and denaturation [30]. Chemical damage occurred as aldehydes and carbonyl compounds promote protein cross-linking, which increased network brittleness and degraded hydration capacity [36]. The resultant structural collapse converted bound water into free water, while ice crystal pores form channels that exacerbate water loss, resulting in a higher centrifugal loss, drip loss and cooking loss.

### 3.4. Protein Digestibility of Pork In Vitro Digestion

Protein digestibility is identified as a key factor influencing the nutritional value and bioavailability of amino acids in meat products [37]. As shown in Table 1, pork subjected to frozen storage exhibited a progressive decline in its susceptibility to pepsin and sequential pepsin–trypsin digestion. Compared to fresh meat, samples that had been frozen for a full year demonstrated the lowest digestibility, with statistically significant reductions (*p* < 0.05) of 11.31% and 11.33% for pepsin and pepsin–trypsin digestibility, respectively. These findings indicated that prolonged freezing substantially diminished protein accessibility during both the gastric and intestinal phases of digestion. The impairment of digestibility was attributed to the cumulative detrimental effects of prolonged frozen storage on the structure of meat. The sustained frozen environment induced protein denaturation and oxidation, which promoted the formation of large, insoluble aggregates through hydrophobic interactions and cross-linking, as previously documented [38]. These aggregates sterically shielded proteolytic cleavage sites, a phenomenon consistent with the established understanding that such conformational changes alter protease recognition sites and reduce enzymatic efficiency [37]. Concurrently, the long-term presence and potential recrystallization of ice, coupled with progressive moisture migration, led to severe and irreversible damage to the muscle microstructure, resulting in tissue hardening [39,40]. This macrostructural collapse created a physical barrier that further restricted the penetration of digestive enzymes to their protein substrates.

The changes in amino acids released during the digestion of protein in the stomach and intestine are important indicators for assessing the digestion characteristics and nutritional quality [38]. The free amino acid content of digestive samples after 120 min of simulated gastrointestinal digestion in vitro was determined. Total free amino acid contents of the protein after simulated gastrointestinal digestion were shown in Table 2. After pepsin hydrolysis, the free amino acid contents of pork protein frozen for 0, 3, and 12 months were 11.63 ± 0.19, 17.63 ± 0.33, and 17.50 ± 0.31 mg/g, respectively, while the free amino acid content after trypsin digestion were 567.0 ± 4.13, 490.88 ± 4.25, and 450.63 ± 10.13 mg/g, respectively. The results indicated that the free amino acid release of pork protein during digestion mainly occurred during trypsin digestion [41].

No significant difference was observed in the total amount of free amino acids between frozen pork protein and fresh pork protein after pepsin digestion (*p* > 0.05). However, there was a significant difference after trypsin digestion (*p* < 0.05). The protein was decomposed into free amino acids and small molecular peptides and was absorbed and utilized by the human body [42]. After digestion of pork protein treated with different freezing time, the content of free amino acids decreased, which destroyed the nutrition of pork. This was consistent with the conclusion that the digestibility of pork protein decreased with the increase in freezing treatment time in previous experiments. The analysis of the release pattern of free amino acids in the hydrolysis process needs to be combined with the subsequent exploration of the potential mechanisms affecting enzymatic digestion.

### 3.5. Microstructure of Protein

**Figure 2:**
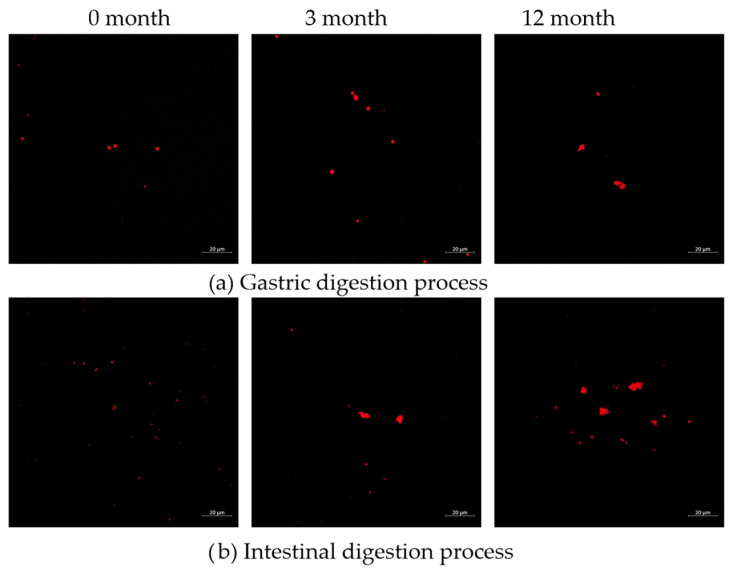
Microstructure of gastric and intestinal digestion product in pork. (**a**) Gastric digestion process; (**b**) Intestinal digestion process.

**Figure 4:**
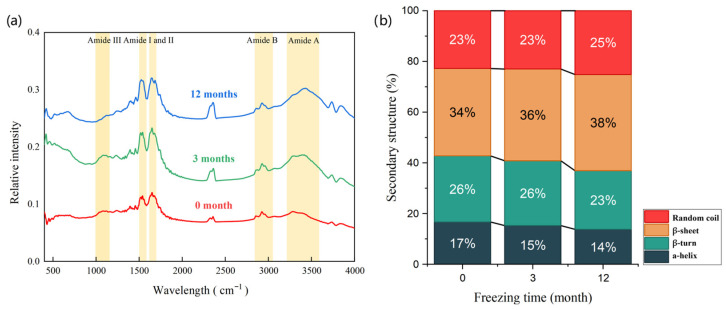
FTIR spectroscopy of pork digestion products treated with different freezing time. (**a**) Relative intensity of gastric digestion product; (**b**) Relative contents of secondary structure of gastric digestion product.

CLSM provided direct visualization of how frozen storage duration altered the structure of protein digestion under simulated gastrointestinal conditions. As illustrated in Figure 2, microstructural analysis quantified by particle size distribution demonstrated a consistent increase in both the dimensions and number density of Nile blue-stained protein aggregates during both gastric and intestinal digestion phases. In fresh samples, meat proteins were initially hydrolyzed by pepsin into polypeptides, which were subsequently efficiently degraded into smaller peptides during intestinal processing. This sequential breakdown pattern confirmed the superior proteolytic efficiency of fresh samples, consistent with the previous findings [43]. Frozen samples displayed markedly larger protein aggregates during gastric digestion compared to fresh controls. Critically, these oversized supramolecular assemblies persisted through intestinal processing, indicating incomplete digestion. The 12-months frozen samples consistently maintained larger aggregates than both 3-months frozen and fresh samples at equivalent digestion stages, revealing a cumulative detrimental effect of prolonged freezing on proteolytic accessibility. This phenomenon could be explained by protein structural shifts during frozen storage: the disruption of hydrogen bonds leads to protein denaturation and aggregation, which masked protease recognition sites and limits enzymatic attack [44]. The resultant structural changes, particularly the increase in β-sheet content and random coil structures (Figure 4), were known to hinder protease activity [22], ultimately reducing digestibility and leading to the larger, undigested aggregates. These findings align with the mechanistic model proposed by report of Sun et al. [27], wherein cryo-induced protein oxidation and structural denaturation promote aggregation via disulfide cross-linking and enhanced hydrophobic interactions. In minced pork, the freezing process under static conditions at −18 °C was relatively slow, promoting ice crystal formation primarily in the extracellular spaces and leading to osmotic dehydration of the meat particles. Although mincing pre-disrupts muscle fibers, prolonged freezing still promotes moisture migration and local ice recrystallization, which could further damage the already fragmented tissue structure and promote protein denaturation and aggregation.

### 3.6. SDS-PAGE Revealed Protein Aggregation and Cross-Linking

**Figure 3:**
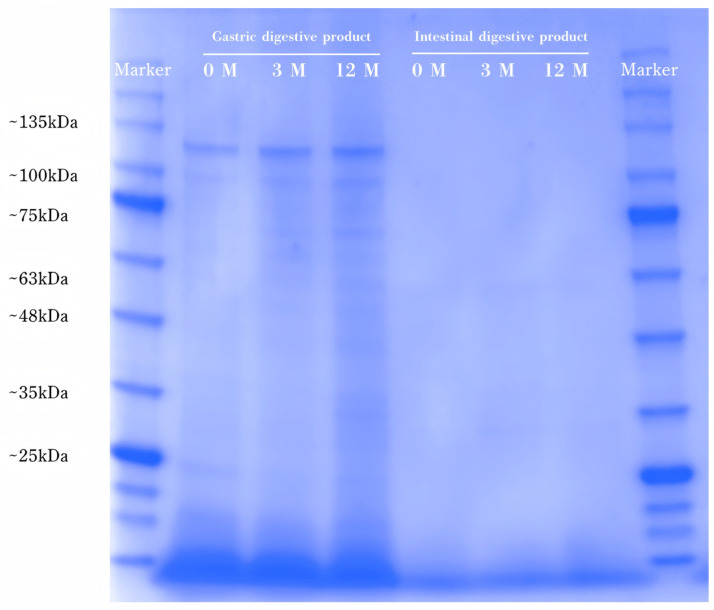
SDS-PAGE results of the expression of protein in digestion product.

SDS-PAGE analysis revealed distinct protein aggregation and degradation patterns in pork samples subjected to varying freezing durations during in vitro digestion. As illustrated in Figure 3, during the gastric phase, high-molecular-weight (HMW) protein bands within the range of 100–135 kDa exhibited progressive broadening with extended freezing, consistent with the accumulation of cryo-induced macromolecular aggregates. The band intensity of low-molecular-weight (LMW) polypeptides around 25 kDa markedly diminished, while a new electrophoretic band appeared at approximately 75 kDa in samples frozen for 12 months. These electrophoretic shifts were attributed to oxidative cross-linking and radical-mediated polymerization of LMW proteins, driven by sustained freeze-induced oxidative stress [45]. A distinct protein band detected near 48 kDa in the 12 months samples, putatively identified as actin based on its molecular weight (~45 kDa), suggested progressive cytoskeletal disintegration caused by ice crystal-mediated mechanical damage to myofibrillar structures. Furthermore, the formation of intermolecular disulfide bonds was also implicated in promoting insoluble aggregate formation, which correlated with the observed decline in proteolytic digestibility in long-term frozen samples [10].

After intestinal digestion, the near-complete disappearance of detectable protein bands across all samples confirmed extensive hydrolysis into peptides and amino acids with molecular weights less than 10 kDa. This result aligned with the established proteolytic mechanism whereby pepsin-catalyzed cleavage in the stomach exposes hydrophobic domains, thereby enhancing trypsin binding and catalytic efficiency in the small intestine, ultimately facilitating the production of absorbable nitrogenous fragments. These results demonstrated that prolonged freezing storage significantly exacerbated protein oxidation and polymerization, ultimately reducing proteolytic accessibility and nutritional bioavailability [46].

### 3.7. Freezing Induced Secondary Structural Changes

FTIR spectroscopy was employed to characterize the secondary structure changes in digestive products, by analyzing the amide I band region (1600–1700 cm^−1^) [47]. The relative contents of specific secondary structures were quantified based on the following spectral intervals: α-helix (1650–1660 cm^−1^), β-sheet (1600–1640 cm^−1^), β-turn (1660–1695 cm^−1^) and random coil (1640–1650 cm^−1^) [48]. The results revealed significant impacts of freezing duration on the secondary structure of proteins during gastric digestion, as shown in Figure 4.

With prolonged freezing duration, the spectral peaks exhibited progressive narrowing and sharpening, consistent with previous reported by Wang et al. [49]. Notably, the amide A and amide B bands demonstrated red shifts, while amide I, II, and III bands showed a slight blue shift. These shifts were interpreted as resulting from enzymatic hydrolysis patterns, in which pepsin and pancreatin preferentially cleave peptide bonds associated with aromatic (Phe, Tyr, Trp) and basic (Arg, Lys) residues [22]. This selective cleavage increased the exposure of C=O and N–H groups, thereby altering hydrogen bonding and vibrational frequencies across amide regions. Extended freezing duration induced a progressive conformational reorganization in the protein secondary structures, as evidenced by analysis of the amide I band. Pepsin digestion subsequently led to a structural shift characterized by a decrease in α-helix and β-turn content, which was accompanied by an increase in β-sheet and random coil proportions. These changes suggested that long-term frozen storage promoted protein unfolding and aggregation during digestion, which in turn influenced proteolytic accessibility [50].

After the 12 months in frozen storage, the secondary structure composition of digestive products was substantially reconfigured. This redistribution was attributed to enzymatic hydrolysis-driven unfolding of native protein conformations, which disrupted ordered structures such as α-helix and β-turn, while favoring the formation of β-sheet and random coils. These structural changes correlated directly with proteolytic efficiency, reduced α-helix content and elevated random coil levels were associated with lower digestibility [27], whereas increased β-sheet content exhibited an inverse relationship with hydrolysis rates. Mechanistic analyzes revealed that cryo-induced protein cross-linking and aggregation obstructed protease cleavage sites, particularly those targeting aromatic (Phe, Tyr) and basic (Arg, Lys) residues [51]. Concurrent transitions from α-helix to β-sheet configurations increased surface hydrophobicity and imposed steric hindrance on enzyme–substrate interactions. Furthermore, the stabilization of β-sheet through extensive hydrogen bonding networks reduced trypsin binding affinity by 55–60% compared to native conformations, as reflected by diminished TCA-soluble peptide yields [10].

### 3.8. Mechanism of the Relationship of Digestibility and Freezing Duration

**Figure 5:**
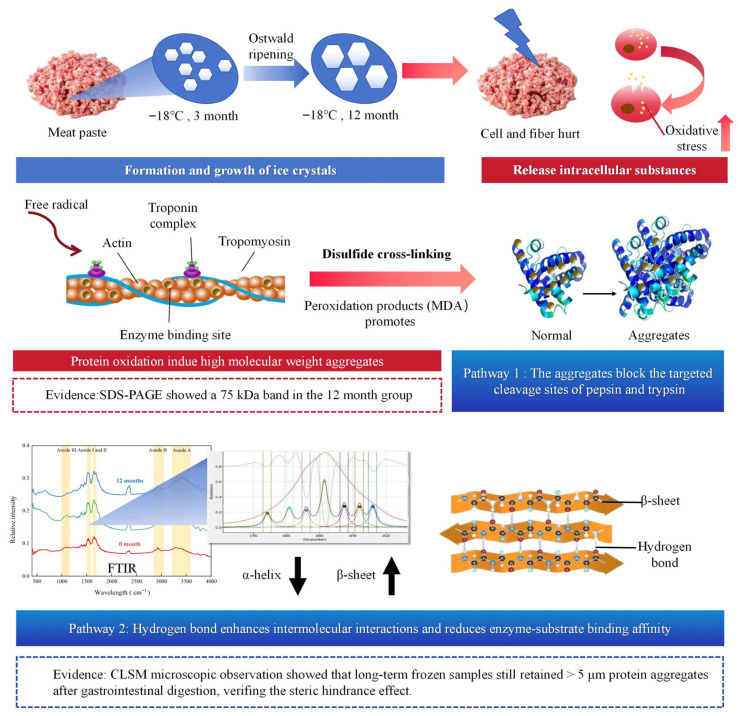
Mechanism of the relationship of digestibility and freezing duration.

The reduction in protein digestibility following long-term freezing was found to stem from a synergistic, multiscale degradation mechanism that integrated physical damage, oxidative modifications, and protein conformational changes [52]. The process was initiated by the physical disruption of muscle tissue, where the growth and fusion of ice crystals through Ostwald ripening pierced cell membranes and myofibrils, which compromised structural integrity and triggered the release of pro-oxidative cellular contents [53]. This created conditions conducive to extensive freezing-induced oxidative cascades, driven by reactive oxygen species (ROS) generated from lipid peroxidation and Fenton reactions [54]. These ROS promoted protein carbonylation and the oxidation of cysteine thiols to disulfide bonds, while lipid peroxidation byproducts such as malondialdehyde formed covalent adducts with lysine residues [54]. These oxidative modifications acted as cross-linking nodes, converting low-molecular-weight proteins into stable high-molecular-weight oligomers (~75 kDa). Concurrently, cryo-denaturation unfolded protein structures, as evidenced by FTIR analysis which showed a 13.78% decrease in α-helix and a concomitant increase in β-sheet (37.90%) and random coil (25.18%), exposed hydrophobic domains and drove further non-covalent aggregation [33]. The resultant dense, cross-linked protein matrix systematically impaired proteolysis through dual pathways (Figure 5): firstly, it created steric hindrance that physically blocked protease access to key cleavage sites for pepsin (Phe/Tyr) and trypsin (Arg/Lys). Secondly, oxidative masking of recognition motifs and the stabilization of β-sheet networks reduced enzyme-binding affinity by 55–60%. The culmination of structural masking, altered surface topology, and restricted peptide bond accessibility was a progressive decline in digestibility, which was directly verified by the persistence of undigested protein aggregates (>5 μm) in CLSM images and an 11.3% drop in digestibility after 12 months. It should be noted that temperature fluctuations during frozen storage, which promote ice recrystallization, could exacerbate protein damage and digestibility reduction. This mechanistic understanding elucidated the fundamental relationship between protein conformational stability and nutritional bioavailability in frozen meat systems, especially household freezing.

The structural unraveling of myofibrillar proteins during prolonged frozen storage led to exposed hydrophobic domains and disrupted enzyme–substrate interactions, ultimately impairing trypsin accessibility to cleavage sites. Consequently, the formation of insoluble protein aggregates directly correlated with reduced in vitro digestibility, underscoring the critical role of cryoprotection in maintaining both structural integrity and nutritional quality.

Notably, this study had a clear limitation in terms of freezing protocol: the experimental design did not adopt industrial shock freezing tunnels operating at around −40 °C, which were commonly used in large-scale meat production. Industrial shock freezing achieved a high cooling rate, rapidly passing the meat through the maximal ice crystallization temperature range to form small and uniform ice crystals, thereby reducing protein damage. In contrast, our current freezing method results in relatively slower cooling rates, leading to larger ice crystal formation and more pronounced structural damage. Consequently, our findings were more applicable to non-industrial freezing scenarios (e.g., household freezing) and could not be directly generalized to large-scale industrial production. Future studies would focus on comparing industrial shock freezing with the current freezing regime to clarify their differences in preserving meat quality and protein digestibility. Furthermore, while the selected storage duration (0, 3, and 12 months) captured relevant practical stages, future experimental designs would benefit from employing more evenly spaced time points (e.g., 0, 3, 6, 9, and 12 months) to better model the kinetic progression of protein structural changes and digestibility loss during frozen storage.

## 4. Conclusions

This study demonstrated that prolonged frozen storage significantly compromised the texture and nutritional value of pork by inducing detrimental changes in protein structure, which in turn impaired its digestibility. As the frozen storage duration increased, the hardness, chewiness, and shear force of the meat significantly increased, while the in vitro protein digestibility decreased. Structural analyzes confirmed that freezing induced protein denaturation and aggregation, which was evidenced by the reduction in α-helix content, an increase in β-sheet and random coil, and the formation of high-molecular-weight aggregates. These changes sterically hindered protease access to cleavage sites and reduced enzyme-binding affinity, ultimately explaining the observed decline in digestibility. The negative impacts were cumulative and intensified with freezing time. These findings underscored that frozen storage duration was a critical factor affecting the protein quality and nutritional value of meat.

## Figures and Tables

**Table 1:** Gastric and intestinal protein digestibility of pork.

Freezing Time /Month	Protein Digestibility/%	
	Gastric	Intestinal
0	25.80 ± 3.56 ^a^	47.56 ± 0.87 ^a^
3	18.27 ± 2.78 ^b^	35.92 ± 0.95 ^b^
12	14.49 ± 0.52 ^c^	33.23 ± 1.24 ^c^

Note: Data were illustrated in mean ± SD (standard deviation), different superscript letters represented significant differences (*p* < 0.05) in each treatment (n = 6).

**Table 2:** Analytical results of free amino acid in digestive products.

Freezing Time (Month)	Contents of Free Amino Acid (mg/g)	
	Gastric Digestion Process	Intestinal Digestion Process
0	11.63 ± 0.19 ^a^	567.0 ± 4.13 ^a^
3	17.63 ± 0.33 ^a^	490.88 ± 4.25 ^b^
12	17.50 ± 0.31 ^a^	450.63 ± 10.13 ^c^

Note: Means with standard error were indicated, different superscript letters represented significant differences (*p* < 0.05) in each treatment (n = 6).

## Data Availability

The original contributions presented in the study are included in the article/Supplementary Materials, further inquiries can be directed to the corresponding authors.

## Supplementary Materials

The following supporting information can be downloaded at: https://www.mdpi.com/article/10.3390/foods15020329/s1, Table S1: Volume of 400 mL (1.25 × concentration) of digestive solution electrolyte reserve solution diluted with UP water; Table S2: Preparation of enzyme stock solution; Table S3: Preparation of digestion master mix.
