# Supplementary material for: Effect of Prolonged Frozen Storage on the In Vitro Digestion of Minced Pork: Insights from Protein Structural Changes
Source: Foods. 2026 Jan 16;15(2):329. doi: 10.3390/foods15020329 (PMC12841090; doi:10.3390/foods15020329)
Supplement: Supplementary file 1 [file foods-15-00329-s001.zip › foods-4062546-supplementary.pdf]

Table S1. Volume of 400 mL (1.25 × concentration) of digestive solution electrolyte reserve solution diluted with UP water.

Salt solution	Concentration	SSF (pH=7)		SGF (pH= 3)		SIF (pH=7)	
	M	mL	mM	mL	mM	mL	mM
KCl	0.5	15.1	15.1	6.9	6.9	6.8	6.8
$KH2PO_4$	0.5	3.7	3.7	0.9	0.9	0.8	0.8
$NaHCO_3$	1	6.8	13.6	12.5	25	42.5	85
NaCl	2	—	—	1.8	47.2	9.6	38.4
$MgCl_2 (H_2O)_6$	0.15	0.5	0.15	0.4	0.12	1.1	0.33
$(NH4)_2CO_3$	0.5	0.06	0.06	0.5	0.5	—	—
HCl	6	0.09	1.1	1.3	15.6	0.7	8.4
$CaCl_2 (H_2O)_2$	0.3	0.025	1.5	0.005	0.15	0.04	0.6

The use of carbonates in electrolyte solutions requires the use of sealed containers with limited space at the top.

Table S2. Preparation of enzyme stock solution

	Mixing ratio	pH	Enzyme activity in the food-fluid mixture	$CaCl_2$ conc
Oral	Food : SSF (w/w)=1:1	7	Amylase 75 U/mL	0.75 mM
Gastric	Bolus : SGF (v/v)=1:1	3	Pepsin 1000 U/mL	0.075 mM
Intestinal	Chyme : SIF (v/v)=1:1	7	Pancreatin 200 U/mL<br>Bile salt 20 mM	0.30 mM

Table S3. Preparation of digestion mastermix

Mastermix	SSF/SGF/SIF	UP Water	0.3M $CaCl_2$	6M HCl	5M NaOH	Enzyme stock
	mL	mL	μL	mL	mL	mL
Oral	12	2.925	75	—	—	5
Gastric	24	1.935	15	1.05	—	5
Intestinal	25.5	11.82	120	—	0.06	2.5
